# Hirschsprung Disease Diagnosis: Calretinin Marker Role in Determining the Presence or Absence of Ganglion Cells

**Published:** 2016

**Authors:** Nasser Rakhshani, Mohammadreza Araste, Farid Imanzade, Mahshid Panahi, Fahimeh Safarnezhad Tameshkel, Masoud Reza Sohrabi, Mohammad Hadi Karbalaie Niya, Farhad Zamani

**Affiliations:** 1 *Gastrointestinal and Liver Diseases Research Center, Firoozgar Hospital, Iran University of Medical Sciences, Tehran, Iran*; 2 *Dept. of Pathology, Iran University of Medical Sciences, Tehran, Iran*; 3 *Dept. of Pediatrics, Shahid Beheshti University of Medical Sciences, Tehran, Iran*; 4 *Dept. of Virology, Iran University of Medical Sciences, Tehran, Iran*

**Keywords:** Hirschsprung disease, Calretinin, Immunohistochemistry, ganglion cell, intrinsic nerve fibers

## Abstract

**Background:**

Hirschsprung disease is a complex genetic disorder of the enteric nervous system (ENS), often called congenital aganglionic megacolon and characterized by the absence of enteric neurons along a variable length of the intestine. The definitive diagnosis of Hirschsprung disease relies on histologic and/or histochemical staining of sections from suction rectal biopsies. Calretinin immunohistochemistry (IHC) may be a useful in its diagnosis. This study aimed to proof the usefulness of immunohistochemical staining for calretinin in rule out of Hirschsprung disease.

**Methods:**

Paraffin blocks and slides were retrieved from the pathology archives of Ali Asghar Hospital, Tehran, Iran from 2007 to 2011 with pathology report based on the presence (14 patients) or absence (70 patients) of ganglion cells and transitional zone anatomical region (10 patients). Slides were stained with hematoxylin and eosin method to confirm the initial diagnosis was verification again. After preparing the slides, they were stained by IHC for calretinin. Then, the results were analyzed using SPSS software.

**Results:**

In most patients, IHC for calretinin provided highly compatible results with hematoxylin-eosin findings in diagnosis of Hirschsprung disease. The values of specificity and accuracy between calretinin and standard histology (H&E) compared by the Fisher exact test declared calretinin presented significantly higher specificity and accuracy values than H&E staining (*P* <0.0001).

**Conclusion:**

Calretinin IHC overcomes most of the difficulties encountered using the histology hematoxylin-eosin. Then, IHC for calretinin is a good ancillary method used by pathologists in diagnosis of Hirschsprung disease.

## Introduction

Hirschsprung disease (HD) is a complex genetic disorder of the enteric nervous system (ENS), often called congenital aganglionic megacolon. The disease is characterized by absence of parasympathetic intrinsic ganglion cells in the distal rectum and a variable length of contiguous bowel (1-4). HD occurs in approximately 1 in 5,000 live births and is one of the more frequent causes of intestinal obstruction in the newborn (5, 6). In most cases of HD (80%), the aganglionic segment involves only the rectum and the sigmoid colon (Short segment aganglionosis), whereas in 20% of cases, the aganglionic segment involves the more proximal bowel (Long segment aganglionosis), and in rare cases may even affect the entire colon (Total colonic aganglionosis) (2, 7, 8). 

Patients who present with HD are usually newborns or infants, thus particular diligence is required in both the diagnosis and treatment. Treatment options include surgical treatment with resection of the aganglionic segment and reconstitution of the intestinal passage after the first year of life, following bridging therapy with colostomy (9). HD is diagnosed histopathologically based on absence of intrinsic ganglion cells from the distal rectum and a variable length of contiguous proximal intestine (5, 10). The rectal suction biopsy (RSB) considered the current gold standard diagnostic test; however, clinical and radiologic correlations are also important. Hematoxylin-eosin (H&E) is commonly used in the diagnosis of HD and most widely relied upon stain in evaluating RSBs (11-14). Despite the importance of using Hematoxylin-eosin and rectal suction biopsy in the diagnosis of HD, diagnosis is impossible with these methods every times. These techniques have limitations, such as morphologic immaturity of ganglion cells particularly in neonates and infants, and that these cells in sub-mucosal area are small and irregularly distributed, so their identification is difficult and requires high expertise (5, 15, 16). Another tool is to identification of aganglionosis and the presence of an increase in acetylcholinesterase-positive hypertrophic nerve fibers in the large bowel submucosa. However, acetylcholinesterase staining is laborious and requires a skilled technician (9) is not universally employed, in part because it requires special tissue handling (17). For this reason, calretinin immunohistochemistry (IHC) may be a useful alternative to confirm the diagnosis of HD (5, 15, 16).

Immunohistochemistry (IHC) is the major supplementary method to identify ganglion cells and nerve hypertrophy. Calretinin, is a calcium binding protein that present in intrinsic nerves of the muscularis mucosae and lamina propria, and can be used as a simple and reliable tool for the diagnosis of HD. Calretinin IHC may be a useful in diagnosis of HD, because loss of calretinin immunoreactive in the ganglion cells or nerve fibers reportedly correlates spatially with aganglionosis. Calretinin IHC has several advantages such as it uses paraffin embedded biopsies, is simple and has a high specificity and sensitivity (2, 18, 19). 

According to the limitations of using hematoxylin - eosin and rectal suction biopsy, and importance of IHC in the diagnosis of immature ganglion cells, this study wanted to show, usefulness of immunohistochemical staining evaluation for calretinin in rule out of HD.

## Materials and Methods

Patients: 

In this cross sectional study, paraffin blocks and slides from 94 patients suspected to HD during the period 2007 to 2011 were retrieved from the pathology archive of Ali Asghar Hospital, Tehran, Iran.

 Informed consent was obtained from each patient. This study has been approved by the Ethics Committee of Iran University of Medical Sciences, Tehran, Iran.

 The paraffin blocks and slides were with pathology report based on the presence (14 patients) or absence (70 patients) of ganglion cells and transitional zone anatomical region (10 patients). The transitional zone anatomical region was defined as the segment between the contracted aganglionic segment and the normal or dilated ganglionated bowel. Skeletal muscle fibers or squamous epithelium represents a sampling from this area. Because there is ensure the absence of ganglion cells in the transitional zone anatomical region, it was used to evaluate the sensitivity of IHC for identification of ganglion cells. Diagnostic criteria for HD in rectal mucosal biopsy are absent ganglion cells and presence of hypertrophic nerve bundles in sub mucosa. Diagnostic criteria for non-HD in rectal mucosal biopsy: At least one ganglion cell is identified in one or more tissue sections.

Hematoxylin- eosin staining and calretinin Immunohistochemistry slides were stained with hematoxylin and eosin method to confirm the initial diagnosis was verification again. Immunostaining was performed on paraffin blocks were retrieved from the pathology archive following the Avidin- Biotin peroxidase technique. 

**Calretinin Immunohistochemistry staining:**

The slides for IHC were processed as follows: First sections of 4 μm were obtained and fixed on the slides with polyelizine. Slides were dewaxed and rehydrated next; they were incubated with the primary monoclonal antibody (Dako, Carpintera, Clifornia, clone DAK Calret 1). 

Following Immunostaining, the slides were examined for ganglion cells or INFs. Calretinin immunoreactivity and pattern of staining for ganglion cells (nuclear and cytoplasmic) and INFs (granular) were evaluated in IHC stained slides. 

**Statistical analyses:**

Statistical calculations were carried out using SPSS version 20 software (Chicago, IL, USA). The values for accuracy, specificity, sensitivity, positive predictive value, and negative predictive value were estimated for calretinin IHC when compared with conventional histopathology (H&E).

## Results

**Patient characteristics:**

**Fig 1:**
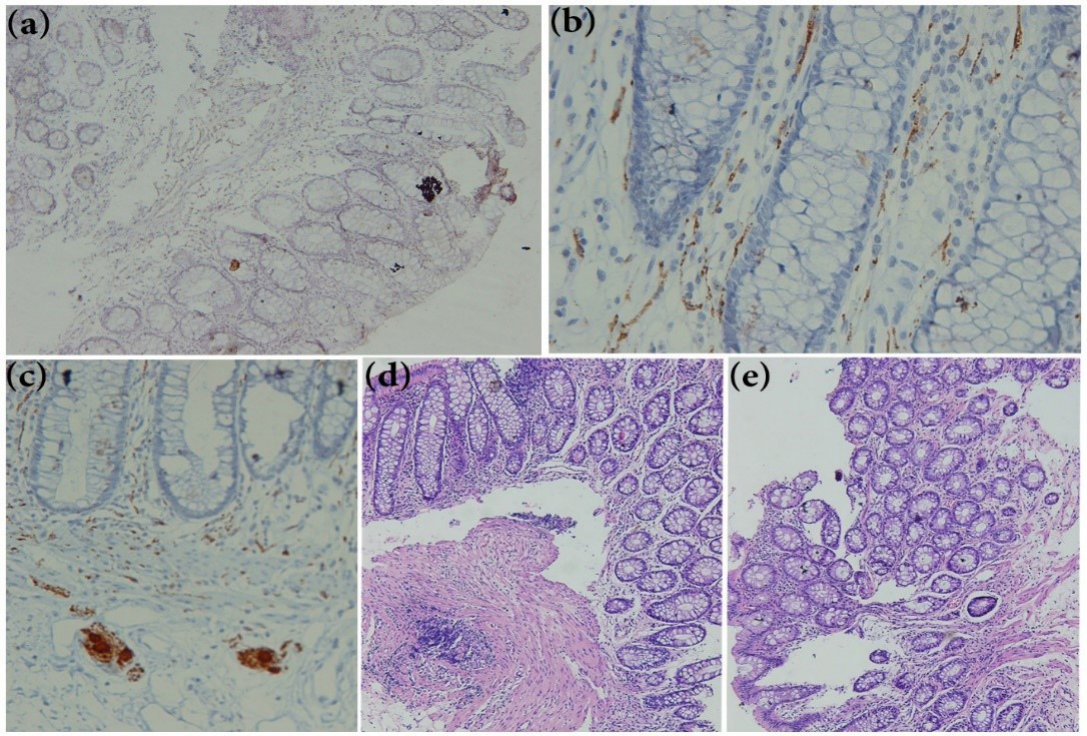
**Rectal biopsy sections of suspicion patient for Hirschsprung disease (HD).** (a) Showing negative reaction after calretinin immunohistochemistry staining (×250). (b) Calretinin positive immunohistochemistry on a normal rectal biopsy sections extended to the lamina propria easily detectable at ×400 magnification in submucosae. (c) Calretinin positive Immunoreactivity in Ganglion cells of submucosa (×400). (d, e) H&E staining rectal biopsy of (d) normal tissue and (e) HD patient

Ninety-four patients including 63 (67.1%) male and 31 (32.9%) female subjects with mean age of 14.37± 22.88 yr (range, one day to 12 yr) months were enrolled. The studied patients were with pathology report based on the presence (14 patients) or absence (70 patients) of ganglion cells and transitional zone anatomical region (10 patients) (Figure 1). 

**Calretinin Immunohistochemistry:**

In the microscopic study of 94 specimens, H&E staining revealed absence of ganglion cells (negative) in 14 cases (14.9%) and presence of cells (positive) in 80 cases (85.1%). In the specimens of the transitional zone anatomical region (An area considered aganglionic), calretinin IHC results in the identification of ganglion cells and intrinsic nerve fibers (INFs) were consistent with the results of H&E staining, except in one sample. Altogether, in the study of specimens through IHC staining with calretinin, of 80 cases were detected negative through H&E, 77 (96.2%) and 76 (95.0%) cases were confirmed in terms of absence of ganglion cells and INFs, respectively (Table 1).

**Table 1:** Detection of Ganglion Cells and INFs in H&E and Calretinin IHC staining

	IHC	H&E		Kappa	*P* value
		Positive (%)	Negative (%)		
Ganglion Cells	Positive	11 (78.6)	3 (21.4)	0.748	<0.0001
	Negative	3 (3.8)	77 (96.2)		
Intrinsic Nerve Fibers (INFs)	Positive	10 (71.4)	4 (28.5)	0.664	<0.0001
	Negative	4 (5.0)	76 (95.0)		
IHC: Immunohistochemistry, H&E: Hematoxylin-eosin					

**Correlation between calretinin IHC and H&E staining:**

Correlation between H&E staining and calretinin IHC in identification of ganglion cells and INFs were calculated by SPSS software which for Ganglion Cells were specificity: 96.2% (77/80), accuracy: 93/6% (88/94), sensitivity: 78.6% (11/14), positive predictive value: 78.6% (11/14), negative predictive value: 96.2% (77/80) and for infs were calculated as specificity: 95% (76/80), accuracy: 91.4% (86/94), sensitivity: 71.4% (10/14), positive predictive value: 71.4% (10/14), and negative predictive value: 95% (76/80), respectively.

Disagreement between calretinin IHC and H&E staining in identification of ganglion cells occurred in 3 cases, whereas disagreement between calretinin IHC and H&E staining in identification of INFs occurred in 4 cases. Comparing the values of specificity and accuracy between calretinin and standard histology (H&E), by the Fisher exact test, calretinin presented significantly (*P *value <0.0001) higher specificity and accuracy values than H&E staining. The measure of agreement by Kappa test showed there were high agreement in IHC and H&E staining of Ganglion Cells and it was significant rather than with INFs (*P *value <0.0001). 

## Discussion

HD is a genetically and phenotypically heterogeneous disorder characterized by complete functional obstruction and colonic dilatation proximal in the large bowel due to the absence of ganglion cells. The aganglionic segment is due to failure of migration of neural crest cells (precursors of enteric ganglion cells) during organogenesis (20-22).

The diagnosis of HD is usually based on a combination of the presenting symptoms, the radiological study, rectal manometry, and histological features of rectal biopsy (23). Despite the importance of using H&E and rectal suction biopsy in the diagnosis of HD, detection of ganglion cells in H&E sections can be a difficult process for the pathologist. The relatively undifferentiated and non-neuronal appearance of immature ganglion cells that exist in the sub-mucosa of neonates and infants is frequently cited as a difficulty associated with H&E-based diagnosis of HD. Accordingly, immunohistochemical (IHC) stains are used to confirm the diagnosis of HD by some pathologists (5, 15, 16). 

Recently, several reports have described the use of calretinin IHC in determining the characteristics of neural distribution in HD. Calretinin was not expressed in aganglionic segments of HD and was associated with nerve fibers whereas both ganglion cells and nerve fibers were immunopositive in normal colons, and was the preferred marker in identification of ganglion cells or intrinsic nerve fibers (INFs) by IHC (18). Calretinin IHC provided highly compatible results with H&E findings in HD (2). Calretinin interpretation was much easier compared with acetylcholinesterase for the junior pathologist (14). “Calretinin IHC overcomes most of the difficulties encountered using the combination of histology and acetylcholinesterase staining” (14).

The present study also showed that results of the calretinin IHC in identification of ganglion cells and INFs are very comparable with H&E staining results. In this study, results of the calretinin IHC in rule out of HD were consistent with the results of H&E staining, except in a few samples. These samples were related to neonates less than 5 months of age. This can be confirmed that maturation of ganglion cells is incomplete at the time of birth, especially in the sub mucosal area, and therefore identification of these cells can be difficult by H&E staining.

Positive calretinin immunostaining was seen in ganglion cells, and calretinin immunostaining in the INFs also represents the presence of related ganglion cells. Even in the absence of ganglion cells, positive immunostaining in INFs is indicating the presence of ganglion cells, and on the other hand, the lack of calretinin-immunoreactivity in INFs confirming the absence of ganglion cells. Finally, comparing the values of specificity and accuracy between calretinin and standard histology (H&E) showed that calretinin IHC presented significantly higher specificity and accuracy values than H&E staining.

 While use of calretinin immunohistochemistry in evaluation for HD appears to be increasing, the published peer-reviewed scientific literature of this technique remains relatively sparse, and a recent standard textbook of Pediatric Pathology comments that this technique may indeed represent a valuable diagnostic adjunct if the utility of (the calretinin) staining pattern can be confirmed by other authors (24).

## Conclusion

Calretinin IHC is a very reliable adjunctive test in identification of ganglion cells and INFs and consequently in ruling out of HD and IHC compared to H AND E stained sections. In addition, calretinin IHC overcomes most of the difficulties encountered using the of histology hematoxylin-eosin. Although limitation of this study included the small population, the results trigger the needs for more investigations in this area and by larger populations. We promise to use calretinin IHC in protocol of HD diagnosis in our institute. Study for other immunohistochemical marker for diagnosis of HD can improve diagnostic approach of HD disease. 
